# Crystal structure of diiso­propyl­aminium di­chloro­acetate

**DOI:** 10.1107/S2056989015007586

**Published:** 2015-04-30

**Authors:** Wei Sun, Guangzhi Shan

**Affiliations:** a Institute of Medicinal Biotechnology, Chinese Academy of Medical Sciences and Peking Union Medical College, Tiantan Xili 1#, Beijing, People’s Republic of China

**Keywords:** crystal structure, diiso­propyl­amine di­chloro­cacetate, hydrogen bonding

## Abstract

In the title compound, C_6_H_16_N^+^·C_2_HCl_2_O_2_^−^, the cation exhibits non-crystallographic *C*_2_ symmetry. In the crystal, the components are linked by N—H⋯O and C—H⋯O hydrogen bonds into chains propagating along [010].

## Related literature   

For the background to the biological activity of the title compound, see: Gelernt & Herbert (2009 ▸); Yamane *et al.* (2014 ▸); Liu *et al.* (2015 ▸). For a related structure, see: Yu & Qian (2009 ▸).
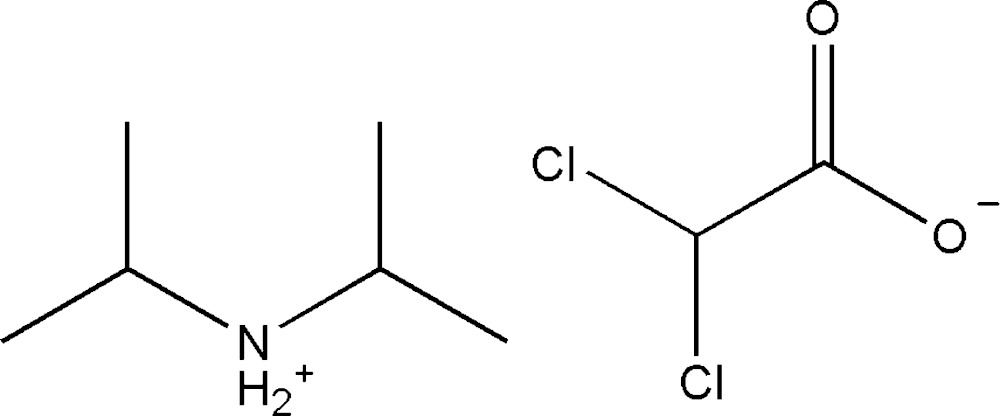


## Experimental   

### Crystal data  

C_6_H_16_N^+^·C_2_HCl_2_O_2_^−^
*M* *_r_* = 230.13
Monoclinic, 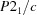

*a* = 10.0272 (2) Å
*b* = 9.04914 (17) Å
*c* = 13.6496 (3) Å
β = 106.433 (2)°
*V* = 1187.94 (4) Å^3^
*Z* = 4
Cu *K*α radiation
μ = 4.71 mm^−1^
*T* = 120 K
0.36 × 0.28 × 0.24 mm

### Data collection  

Agilent Xcalibur Atlas Gemini ultra diffractometer
Absorption correction: multi-scan (*CrysAlis PRO*; Agilent, 2014 ▸) *T*_min_ = 0.662, *T*_max_ = 1.000
11039 measured reflections
2107 independent reflections
1911 reflections with *I* > 2σ(*I*)
*R*_int_ = 0.026

### Refinement  

*R*[*F* ^2^ > 2σ(*F* ^2^)] = 0.038
*wR*(*F* ^2^) = 0.092
*S* = 1.03
2107 reflections
122 parameters
H-atom parameters constrained
Δρ_max_ = 0.81 e Å^−3^
Δρ_min_ = −0.85 e Å^−3^

### 

Data collection: *CrysAlis PRO* (Agilent, 2014 ▸); cell refinement: *CrysAlis PRO*; data reduction: *CrysAlis PRO*; program(s) used to solve structure: *SHELXS97* (Sheldrick, 2008 ▸); program(s) used to refine structure: *SHELXL97* (Sheldrick, 2008 ▸); molecular graphics: *SHELXTL* (Sheldrick, 2008 ▸); software used to prepare material for publication: *OLEX2* (Dolomanov *et al.*, 2009 ▸), *SHELXTL*, *PLATON* (Spek, 2009 ▸) and *publCIF* (Westrip, 2010 ▸).

## Supplementary Material

Crystal structure: contains datablock(s) I. DOI: 10.1107/S2056989015007586/gk2629sup1.cif

Supporting information file. DOI: 10.1107/S2056989015007586/gk2629Isup2.cml

**Fig. 1.**
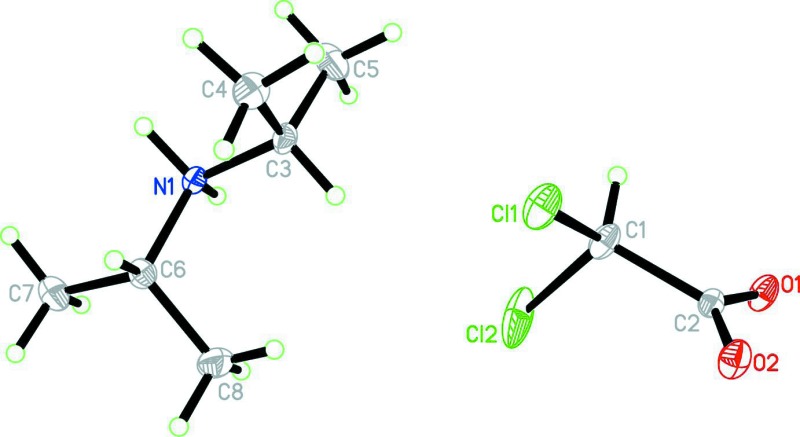
The molecular structure of the title compound. The displacement parameters are shown at the 30% probability level.

. DOI: 10.1107/S2056989015007586/gk2629fig1.tif
The mol­ecular structure of the title compound. The displacement parameters are shown at the 30% probability level.

**Fig. 2.**
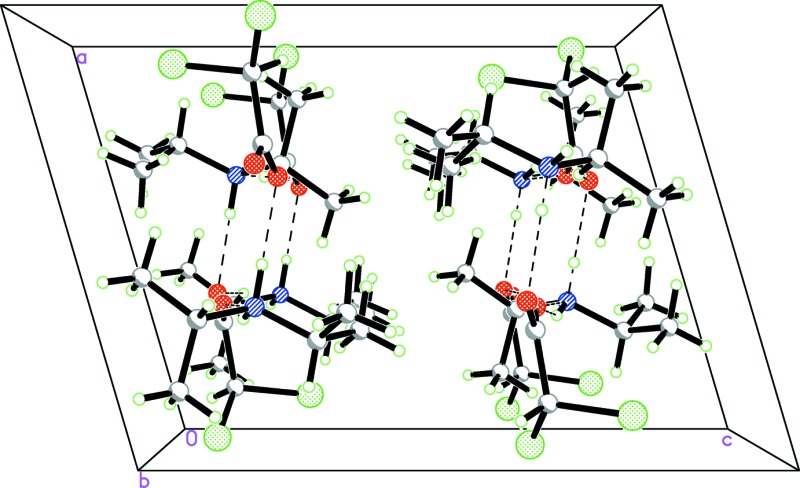
Crystal packing of the title compound, viewed down the b direction. Dashed lines indicate hydrogen bonds.

. DOI: 10.1107/S2056989015007586/gk2629fig2.tif
Crystal packing of the title compound, viewed down the b direction. Dashed lines indicate hydrogen bonds.

CCDC reference: 1060114

Additional supporting information:  crystallographic information; 3D view; checkCIF report

## Figures and Tables

**Table 1:** Hydrogen-bond geometry (, )

*D*H*A*	*D*H	H*A*	*D* *A*	*D*H*A*
N1H1*A*O1^i^	0.92	1.87	2.788(2)	177
N1H1*B*O2^ii^	0.92	1.90	2.757(2)	154
C6H6O1^iii^	1.00	2.38	3.258(2)	146

Symmetry codes: (i) 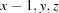; (ii) 
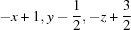
; (iii) 
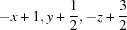
.

## supplementary crystallographic information

### S1. Comment

Diisopropylaminium dichlorocacetate (DADA) is the effective constituent of Vitamin B15 (Gelernt *et al.*, 2009). Recently, DADA has been found as a potential PDK4 inhibitor for treatment of severe influenza (Yamane *et al.*, 2014). Moreover the desirable therapeutic effects of colorectal cancer in mat is shown (Liu *et al.*, 2015). No data about the crystal structure of diisopropylaminium dichlorocacetate has been reported so far.

The title molecule is shown in Fig. 1. The asymmetric unit contains one diisopropylaminium cation and one dichloroacetate anion. In the crystal structure, the cations and anions are linked by intermolecular N—H···O and C—H···O hydrogen bonds (Table 1, Fig.2) to form chains propagating along [010].

### S2. Experimental

Single crystals suitable for X-ray analysis were obtained by slow solvent evaporation from a solution of the title compound in a dichloromethane/cyclohexane mixture (1:1 *v*/*v*) at room temperature.

### S3. Refinement

All H atoms were positioned geometrically and constrained to ride on the parent atoms, with N—H = 0.92 Å and C—H = 0.98–1.00 Å, and with *U*_iso_(H) = 1.5*U*_eq_(methyl C) or 1.2*U*_eq_(C, N) for other H atoms.

### Crystal data

**Table:**

C_6_H_16_N^+^·C_2_HCl_2_O_2_^−^	*F*(000) = 488
*M**_r_* = 230.13	*D*_x_ = 1.287 Mg m^−^^3^
Monoclinic, *P*2_1_/*c*	Cu *K*α radiation, λ = 1.54184 Å
*a* = 10.0272 (2) Å	Cell parameters from 5039 reflections
*b* = 9.04914 (17) Å	θ = 4.6–66.9°
*c* = 13.6496 (3) Å	µ = 4.71 mm^−^^1^
β = 106.433 (2)°	*T* = 120 K
*V* = 1187.94 (4) Å^3^	Block, colourless
*Z* = 4	0.36 × 0.28 × 0.24 mm

### Data collection

**Table:**

Agilent Xcalibur Atlas Gemini ultra diffractometer	2107 independent reflections
Radiation source: sealed X-ray tube, Enhance Ultra (Cu) X-ray Source	1911 reflections with *I* > 2σ(*I*)
Mirror monochromator	*R*_int_ = 0.026
Detector resolution: 10.4674 pixels mm^-1^	θ_max_ = 66.9°, θ_min_ = 4.6°
ω scans	*h* = −11→11
Absorption correction: multi-scan (*CrysAlis PRO*; Agilent, 2014)	*k* = −7→10
*T*_min_ = 0.662, *T*_max_ = 1.000	*l* = −16→16
11039 measured reflections	

### Refinement

**Table:**

Refinement on *F*^2^	Primary atom site location: structure-invariant direct methods
Least-squares matrix: full	Secondary atom site location: difference Fourier map
*R*[*F*^2^ > 2σ(*F*^2^)] = 0.038	Hydrogen site location: inferred from neighbouring sites
*wR*(*F*^2^) = 0.092	H-atom parameters constrained
*S* = 1.03	*w* = 1/[σ^2^(*F*_o_^2^) + (0.0344*P*)^2^ + 1.3398*P*] where *P* = (*F*_o_^2^ + 2*F*_c_^2^)/3
2107 reflections	(Δ/σ)_max_ < 0.001
122 parameters	Δρ_max_ = 0.81 e Å^−^^3^
0 restraints	Δρ_min_ = −0.85 e Å^−^^3^

### Special details

**Table:**

Experimental. The crystal was placed in the cold stream of an Oxford Cryosystems open-flow nitrogen cryostat with a nominal stability of 0.1 K. .
Geometry. All e.s.d.'s (except the e.s.d. in the dihedral angle between two l.s. planes) are estimated using the full covariance matrix. The cell e.s.d.'s are taken into account individually in the estimation of e.s.d.'s in distances, angles and torsion angles; correlations between e.s.d.'s in cell parameters are only used when they are defined by crystal symmetry. An approximate (isotropic) treatment of cell e.s.d.'s is used for estimating e.s.d.'s involving l.s. planes.
Refinement. Refinement of *F*^2^ against ALL reflections. The weighted *R*-factor *wR* and goodness of fit *S* are based on *F*^2^, conventional *R*-factors *R* are based on *F*, with *F* set to zero for negative *F*^2^. The threshold expression of *F*^2^ > σ(*F*^2^) is used only for calculating *R*-factors(gt) *etc*. and is not relevant to the choice of reflections for refinement. *R*-factors based on *F*^2^ are statistically about twice as large as those based on *F*, and *R*- factors based on ALL data will be even larger.

### Fractional atomic coordinates and isotropic or equivalent isotropic displacement parameters (Å^2^)

**Table:**

	*x*	*y*	*z*	*U*_iso_*/*U*_eq_	
Cl1	0.53986 (5)	0.16195 (6)	0.60806 (5)	0.04006 (18)	
Cl2	0.62278 (7)	−0.05527 (9)	0.76920 (6)	0.0651 (3)	
O1	0.86608 (14)	−0.08510 (15)	0.66082 (12)	0.0292 (3)	
O2	0.84614 (15)	0.15792 (15)	0.68081 (12)	0.0308 (3)	
N1	0.15036 (16)	−0.05351 (17)	0.75375 (12)	0.0202 (3)	
H1A	0.0563	−0.0607	0.7230	0.024*	
H1B	0.1801	−0.1441	0.7822	0.024*	
C2	0.80092 (19)	0.0312 (2)	0.66498 (14)	0.0216 (4)	
C3	0.2208 (2)	−0.0234 (2)	0.67193 (15)	0.0268 (4)	
H3	0.3218	−0.0050	0.7050	0.032*	
C6	0.1729 (2)	0.0574 (2)	0.83878 (15)	0.0256 (4)	
H6	0.1401	0.1563	0.8087	0.031*	
C4	0.1577 (2)	0.1123 (3)	0.61147 (17)	0.0331 (5)	
H4A	0.0568	0.0998	0.5855	0.050*	
H4B	0.1972	0.1253	0.5540	0.050*	
H4C	0.1785	0.1995	0.6558	0.050*	
C1	0.6441 (2)	0.0036 (2)	0.65013 (17)	0.0291 (5)	
H1	0.6122	−0.0772	0.5989	0.035*	
C7	0.0847 (3)	0.0110 (3)	0.90727 (17)	0.0362 (5)	
H7A	−0.0130	0.0049	0.8669	0.054*	
H7B	0.0943	0.0840	0.9619	0.054*	
H7C	0.1158	−0.0858	0.9373	0.054*	
C5	0.2054 (3)	−0.1610 (3)	0.60590 (18)	0.0421 (6)	
H5A	0.2486	−0.2451	0.6483	0.063*	
H5B	0.2513	−0.1450	0.5522	0.063*	
H5C	0.1065	−0.1815	0.5747	0.063*	
C8	0.3249 (2)	0.0689 (3)	0.8971 (2)	0.0436 (6)	
H8A	0.3593	−0.0283	0.9247	0.065*	
H8B	0.3363	0.1395	0.9533	0.065*	
H8C	0.3779	0.1029	0.8511	0.065*	

### Atomic displacement parameters (Å^2^)

**Table:**

	*U*^11^	*U*^22^	*U*^33^	*U*^12^	*U*^13^	*U*^23^
Cl1	0.0265 (3)	0.0351 (3)	0.0558 (4)	0.0130 (2)	0.0072 (2)	0.0078 (2)
Cl2	0.0341 (3)	0.0849 (5)	0.0860 (5)	0.0143 (3)	0.0328 (3)	0.0510 (4)
O1	0.0201 (7)	0.0203 (7)	0.0470 (9)	0.0020 (6)	0.0090 (6)	−0.0021 (6)
O2	0.0266 (7)	0.0206 (7)	0.0473 (9)	−0.0036 (6)	0.0139 (6)	−0.0056 (6)
N1	0.0162 (7)	0.0192 (8)	0.0250 (8)	0.0001 (6)	0.0055 (6)	0.0007 (6)
C2	0.0188 (9)	0.0231 (10)	0.0229 (9)	0.0003 (8)	0.0056 (7)	0.0012 (7)
C3	0.0173 (9)	0.0376 (12)	0.0271 (10)	−0.0007 (8)	0.0091 (8)	0.0046 (9)
C6	0.0270 (10)	0.0204 (10)	0.0292 (10)	0.0020 (8)	0.0078 (8)	−0.0026 (8)
C4	0.0333 (12)	0.0334 (12)	0.0334 (11)	−0.0038 (9)	0.0106 (9)	0.0076 (9)
C1	0.0187 (10)	0.0231 (10)	0.0434 (12)	0.0032 (8)	0.0053 (9)	0.0030 (9)
C7	0.0416 (13)	0.0394 (13)	0.0313 (11)	0.0023 (10)	0.0161 (10)	−0.0025 (10)
C5	0.0558 (15)	0.0426 (14)	0.0327 (12)	0.0147 (12)	0.0201 (11)	0.0021 (10)
C8	0.0314 (12)	0.0473 (14)	0.0466 (14)	−0.0040 (11)	0.0020 (10)	−0.0203 (12)

### Geometric parameters (Å, º)

**Table:**

Cl1—C1	1.771 (2)	C6—C8	1.511 (3)
Cl2—C1	1.780 (2)	C4—H4A	0.9800
O1—C2	1.248 (2)	C4—H4B	0.9800
O2—C2	1.230 (2)	C4—H4C	0.9800
N1—H1A	0.9200	C1—H1	1.0000
N1—H1B	0.9200	C7—H7A	0.9800
N1—C3	1.505 (2)	C7—H7B	0.9800
N1—C6	1.503 (2)	C7—H7C	0.9800
C2—C1	1.548 (3)	C5—H5A	0.9800
C3—H3	1.0000	C5—H5B	0.9800
C3—C4	1.514 (3)	C5—H5C	0.9800
C3—C5	1.519 (3)	C8—H8A	0.9800
C6—H6	1.0000	C8—H8B	0.9800
C6—C7	1.516 (3)	C8—H8C	0.9800
			
H1A—N1—H1B	107.3	H4B—C4—H4C	109.5
C3—N1—H1A	108.1	Cl1—C1—Cl2	109.02 (12)
C3—N1—H1B	108.1	Cl1—C1—H1	108.8
C6—N1—H1A	108.1	Cl2—C1—H1	108.8
C6—N1—H1B	108.1	C2—C1—Cl1	113.38 (14)
C6—N1—C3	116.92 (15)	C2—C1—Cl2	107.99 (14)
O1—C2—C1	112.56 (16)	C2—C1—H1	108.8
O2—C2—O1	128.50 (18)	C6—C7—H7A	109.5
O2—C2—C1	118.92 (17)	C6—C7—H7B	109.5
N1—C3—H3	108.9	C6—C7—H7C	109.5
N1—C3—C4	109.89 (16)	H7A—C7—H7B	109.5
N1—C3—C5	107.58 (17)	H7A—C7—H7C	109.5
C4—C3—H3	108.9	H7B—C7—H7C	109.5
C4—C3—C5	112.62 (18)	C3—C5—H5A	109.5
C5—C3—H3	108.9	C3—C5—H5B	109.5
N1—C6—H6	108.7	C3—C5—H5C	109.5
N1—C6—C7	107.75 (16)	H5A—C5—H5B	109.5
N1—C6—C8	111.19 (16)	H5A—C5—H5C	109.5
C7—C6—H6	108.7	H5B—C5—H5C	109.5
C8—C6—H6	108.7	C6—C8—H8A	109.5
C8—C6—C7	111.81 (19)	C6—C8—H8B	109.5
C3—C4—H4A	109.5	C6—C8—H8C	109.5
C3—C4—H4B	109.5	H8A—C8—H8B	109.5
C3—C4—H4C	109.5	H8A—C8—H8C	109.5
H4A—C4—H4B	109.5	H8B—C8—H8C	109.5
H4A—C4—H4C	109.5		
			
O1—C2—C1—Cl1	−157.58 (15)	C3—N1—C6—C7	175.93 (16)
O1—C2—C1—Cl2	81.51 (19)	C3—N1—C6—C8	−61.2 (2)
O2—C2—C1—Cl1	23.8 (2)	C6—N1—C3—C4	−67.1 (2)
O2—C2—C1—Cl2	−97.11 (19)	C6—N1—C3—C5	169.99 (17)

### Hydrogen-bond geometry (Å, º)

**Table:**

*D*—H···*A*	*D*—H	H···*A*	*D*···*A*	*D*—H···*A*
N1—H1*A*···O1^i^	0.92	1.87	2.788 (2)	177
N1—H1*B*···O2^ii^	0.92	1.90	2.757 (2)	154
C6—H6···O1^iii^	1.00	2.38	3.258 (2)	146

Symmetry codes: (i) *x*−1, *y*, *z*; (ii) −*x*+1, *y*−1/2, −*z*+3/2; (iii) −*x*+1, *y*+1/2, −*z*+3/2.

## References

[bb1] Agilent (2014). *CrysAlis PRO*. Agilent Technologies, Yarnton, England.

[bb2] Dolomanov, O. V., Bourhis, L. J., Gildea, R. J., Howard, J. A. K. & Puschmann, H. (2009). *J. Appl. Cryst.* **42**, 339–341.

[bb3] Gelernt, M. D. & Herbert, V. (1982). *Nutr. Cancer*, **3**, 129–133. 10.1080/01635588109513714 6752894

[bb4] Liu, D.-X., Wang, F.-F., Yue, J., Jing, X.-B. & Huang, Y.-B. (2015). *Drug Deliv.* **22**, 136–143. 10.3109/10717544.2013.870258 24359441

[bb5] Sheldrick, G. M. (2008). *Acta Cryst.* A**64**, 112–122. 10.1107/S0108767307043930 18156677

[bb6] Spek, A. L. (2009). *Acta Cryst.* D**65**, 148–155. 10.1107/S090744490804362X PMC2631630 19171970

[bb7] Westrip, S. P. (2010). *J. Appl. Cryst.* **43**, 920–925.

[bb8] Yamane, K., Indalao, I.-L., Chida, J., Yamamoto, Y., Hanawa, M. & Kido, H. (2014). *PLos ONE*, **9**, e98032. 10.1371/journal.pone.0098032 PMC4035290 24865588

[bb9] Yu, Y.-H. & Qian, K. (2009). *Acta Cryst.* E**65**, o1278. 10.1107/S1600536809016626 PMC2969592 21583140

