# Supplementary figures and images for: Crystal structure of diiso­propyl­aminium di­chloro­acetate
Source: Acta Crystallogr E Crystallogr Commun. 2015 Apr 30;71(Pt 5):o361. doi: 10.1107/S2056989015007586 (PMC4420103; doi:10.1107/S2056989015007586)

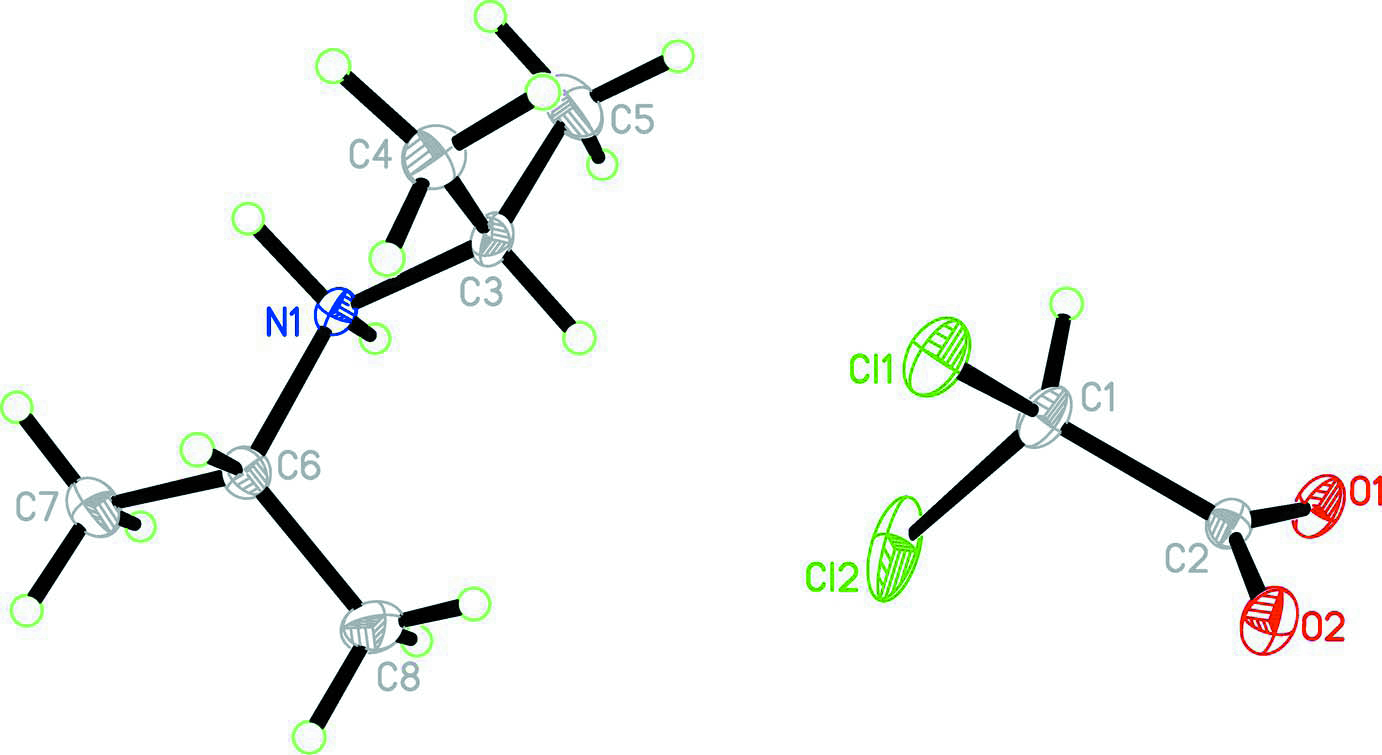

Supplement: Supplementary file 3 [file e-71-0o361-fig1.tif]

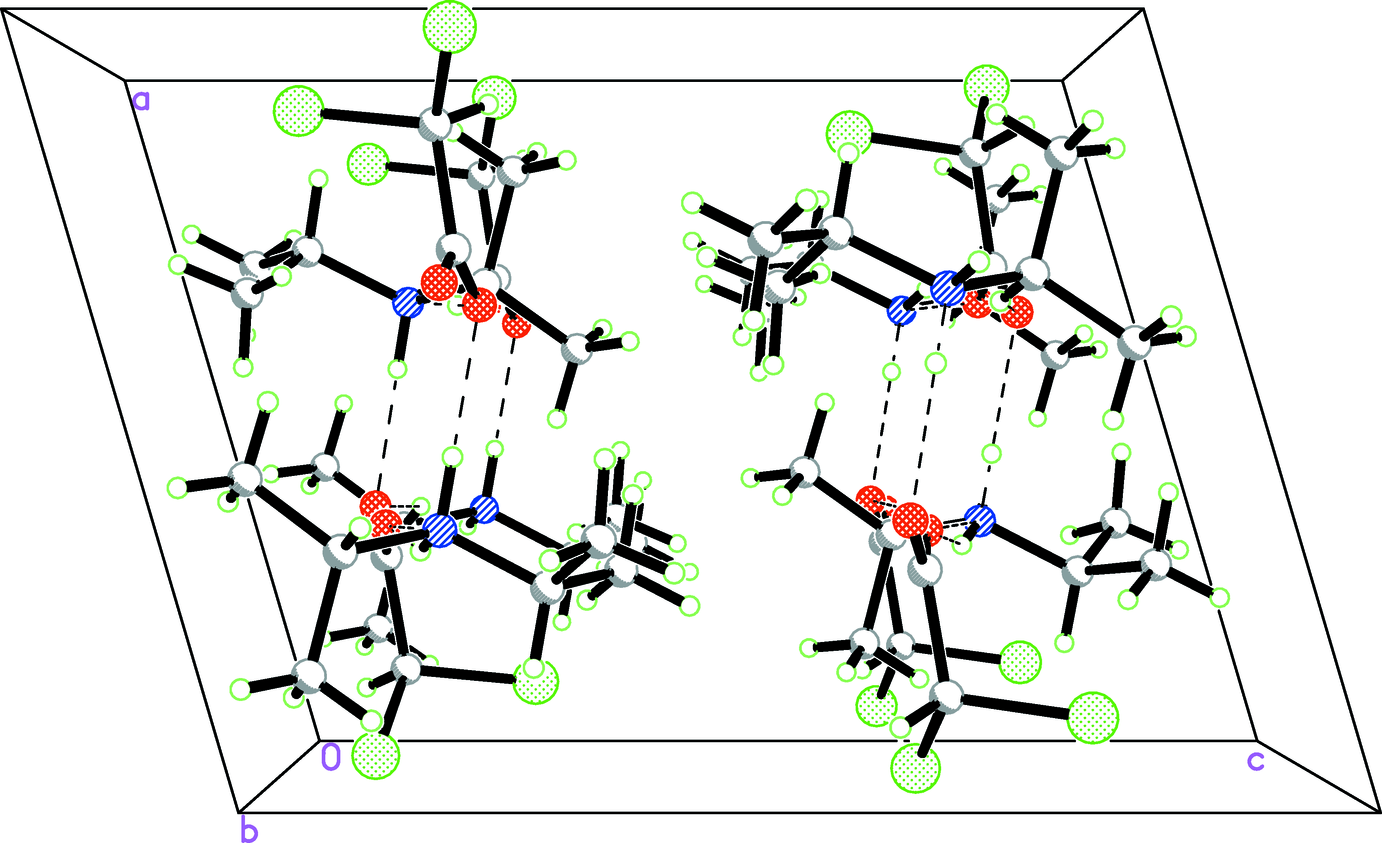

Supplement: Supplementary file 4 [file e-71-0o361-fig2.tif]
